# Symmetry Breaking and Epithelial Cell Extrusion

**DOI:** 10.3390/cells9061416

**Published:** 2020-06-07

**Authors:** Bageshri Naimish Nanavati, Alpha S. Yap, Jessica L. Teo

**Affiliations:** Division of Cell and Developmental Biology, Institute for Molecular Bioscience, The University of Queensland, St. Lucia, Brisbane, QLD 4072, Australia; b.nanavati@uq.edu.au (B.N.N.); j.teo@imb.uq.edu.au (J.L.T.)

**Keywords:** apoptotic extrusion, oncogenic extrusion, contractility, actomyosin

## Abstract

Cell extrusion is a striking morphological event found in epithelia and endothelia. It is distinguished by two symmetry-breaking events: a loss of planar symmetry, as cells are extruded in either apical or basal directions; and loss of mechanochemical homogeneity within monolayers, as cells that are fated to be extruded become biochemically and mechanically distinct from their neighbors. Cell extrusion is elicited by many diverse events, from apoptosis to the expression of transforming oncogenes. Does the morphological outcome of extrusion reflect cellular processes that are common to these diverse biological phenomena? To address this question, in this review we compare the progress that has been made in understanding how extrusion is elicited by epithelial apoptosis and cell transformation.

## 1. Introduction

**Figure 1:**
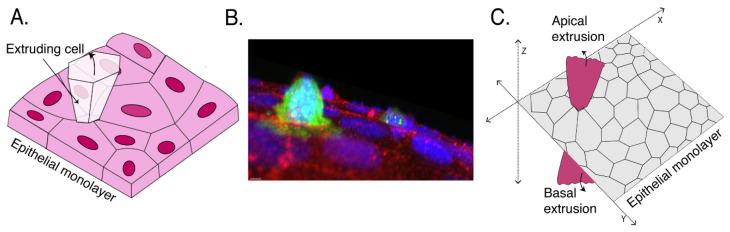
Symmetry breaking in cell extrusion. (**A**) Epithelia face diverse challenges to their integrity and homeostasis, including cell death, transformation, and overcrowding. One striking homeostatic response is for cells to be physically expelled from the monolayer in either an apical or basal direction. This process is called cell extrusion. (**B**) Immunofluorescence image of a cell expressing H-Ras^V12^ being apically extruded from a Caco-2 colon epithelial monolayer. (Green: GFP-H-Ras^V12^; red: N-WASP marking cell–cell contacts; purple: DAPI; courtesy of Dr. Selwin Wu.) (**C**) During the process of cell extrusion, the overall geometric symmetry of tissue is broken as the cell is expelled out in Z dimension either apically or basally. Moreover, the affected cell is biochemically and often mechanically different from its neighboring healthy cells, breaking the planar symmetry or homogeneity of the monolayer.

Cell extrusion is a distinctive morphological phenomenon where cells are physically expelled from tissues. This expulsion process, also described as delamination, is strikingly evident where the affected cells appear to pop out of their tissue of origin (Figure 1A,B). Characteristically, extrusion occurs in epithelia and endothelia tissues [1] which consist of polarized cells linked together by cell–cell junctions. For simplicity, in this article we will principally refer to epithelia, where much of the work has been done to date.

Extrusion involves two forms of symmetry breaking (Figure 1C). First, extrusion breaks the planar symmetry of the host tissue by expelling cells in a direction that is orthogonal to the plane of its monolayer. This can be understood as a form of geometric symmetry breaking (Figure 1C). Cell expulsion has been described to occur in either apical or basal directions, which would generally lead to the expelled cells being directed into the external environment or towards entering the body, respectively. For simplicity, we will focus on apical extrusion in this article. Secondly, it is increasingly evident that important biochemical and biomechanical differences distinguish the cells destined for expulsion from their surrounding neighbors. Thus, at a first generalization we can consider extrusion to disrupt the biochemical and mechanical homogeneity of the tissue at the interface between the extruded cell and its neighbors (Figure 1C). Ultimately, any adequate description of the extrusion process must explain how the geometric event of expulsion arises from the biochemical and mechanical differences between the expelled cell and its neighbors.

A striking feature of the extrusion phenomenon is the diverse range of biological processes in which it has been implicated. For example, it is elicited when epithelial cells undergo apoptosis (apoptotic extrusion [2]) or when they express oncogenes (oncogenic extrusion [3]). Extrusion also occurs when epithelia become overcrowded [1,4] or when they are infected with intracellular organisms, such as salmonella [5,6]. In some of these cases, extrusion can be understood as a way of preserving tissue homeostasis, but in other circumstances extrusion is involved in cell differentiation [7]. This suggests that extrusion may be a final common response to very different biological processes. What is not clear, though, is whether the common morphological features of extrusion reflect biochemical and mechanobiological mechanisms that are shared between these different processes, or whether different forms of extrusion may be better understood as distinct phenomena. In this review, we endeavor to consider this question by comparing what is currently known about the two best-studied forms of extrusion: apoptotic and oncogenic extrusion. 

## 2. Defining Extrusion

To begin, it is useful to consider how we define cell extrusion. The term is often used to refer to the morphological process of expulsion or delamination. This is understandable, given the dramatic morphology of this process. However, diverse mechanisms may be responsible for similar morphological events. For example, the basal egress of transformed cells from an epithelium has often been ascribed to epithelial–mesenchymal transitions and local degradation of the basement membrane [8] as well as to basal extrusion. On purely morphological grounds alone, it is difficult to distinguish what may be mechanistically distinct phenomena.

In addition, to focus on expulsion alone runs the risk of overlooking a key feature of the extrusion process, namely the necessary involvement of the epithelial cells which surround the cell that will be extruded (“neighbor cells” for simplicity). This is exemplified by the role of E-cadherin, the classical cadherin that is a major component of adherens junctions (AJ) in epithelia. Both apoptotic [9] and oncogenic extrusion [3] are inhibited when E-cadherin is depleted in the epithelium surrounding the cells that are to be extruded. Although it is not yet clear why E-cadherin is required in the neighbors, this role for cell–cell interactions between neighbor cells implies that extrusion is a cell nonautonomous phenomenon. The concept of cell nonautonomy in the surrounding epithelium is reinforced by evidence that cell shape [2], the cytoskeleton [10,11], and cell signaling pathways [12] are altered in the neighbor cells—often reflecting the biochemical symmetry breaking described earlier—and these changes may be confined to the local environment of the extruded cells (only one or a few cell diameters away). 

We therefore suggest that it is useful to define extrusion as encompassing both (a) the phenomenon of cell expulsion; and (b) the active contribution of the surrounding monolayer, both to expel the extruded cell and, as we shall see, to preserve the epithelial barrier itself. Taking this perspective, we will frame our review of apoptotic and oncogenic extrusion around the following questions: (1) What are the relevant biochemical and/or mechanical changes that occur in the cells to be extruded? What changes in these cells are responsible for triggering the extrusion process? (2) How do the neighbor cells participate in the extrusion response? And what are the underlying cell biological and mechanical changes that characterize the neighbor response? 3) How are changes in the extruded cells coordinated with changes in their neighbors? Is there cell-to-cell communication between these two cell populations? As we shall see, the answers to these questions are provisional and may not be the same for apoptotic and oncogenic extrusion.

## 3. Apoptotic Cell Extrusion

Apoptosis is a form of programmed cell death and a major cause of tissue turnover during development and in postdevelopmental life [13]. Indeed, apoptosis is estimated to be responsible for the daily turnover of ~150 billion cells in the healthy human body [14]. However, this burden of cell turnover presents a homeostatic challenge for the body: to prevent the release of cellular contents that can provoke inflammatory and autoimmune reactions. In the first instance, this is achieved by apoptotic cells becoming fragmented into membrane-bound apoptotic bodies [15,16]. The immunological inertness of the apoptotic process can be maintained so long as those membranes remain intact. However, membrane integrity can eventually be lost through secondary necrosis [17]. Therefore, additional mechanisms are required to eliminate apoptotic corpses and fragments before their membrane integrity becomes compromised. The best-understood of these secondary mechanisms is the phagocytosis of apoptotic bodies (efferocytosis) by macrophages/monocytes and nonprofessional phagocytes (including epithelial cells) [18,19]. 

However, epithelia and endothelia can also eliminate apoptotic cells by extrusion. This has been observed in model systems [20], but also occurs in pure cell culture [2,11,20] indicating that it is an epithelium-intrinsic process that does not require other cells of the innate immune system. Indeed, apical extrusion has a potential design advantage: since the apical surface of epithelial cells typically faces to the external environment, apical extrusion allows apoptotic cells to be directly eliminated from the body. Of note, extrusion appears to be engaged as a relatively early response to apoptosis: it begins—and is sometimes completed [21]—before markers of late apoptosis (such as the ectofacial presentation of phosphatidylserine) become evident [2,11]. Thus, apical extrusion may constitute a first-line response to apoptosis in epithelia, one that can complement, or even prevent, the need for efferocytosis. It should be noted, though, that there are species-specific differences: whereas apoptotic extrusion typically occurs in an apical direction in vertebrates, in Drosophila, apoptotic cells are commonly extruded basally [22,23,24,25,26,27].

The extrusion process can also address another challenge that apoptosis presents for epithelia. The potential for apoptotic bodies to break down and provoke inflammation is one that is probably shared by all tissues. But epithelia have the additional responsibility of providing major physiological barriers in the body. In particular, epithelia provide the barrier separating the body from its external environment, controlling the flux of ions and fluid, absorbing nutrients, and preventing the entry of infective and noxious agents [28]. Functioning epithelial barriers require both intact cells and also specialized cell–cell junctions, both of which are potentially compromised by apoptosis. Thus, it is striking that epithelial barrier function is preserved even when apoptosis is enhanced by agents such as etoposide [2,9]. Here, the neighbor cell response appears to critically preserve the barriers. During apoptotic extrusion, neighbor cells elongate and extend lamellipodial extensions underneath the apoptotic cell [29,30]. It is thought that these responses allow tight and adhesive junctions to be preserved between the neighbor cells, thereby maintaining the barrier whilst the apoptotic cell is eliminated. Indeed, both AJ and desmosomes are preserved during the process of apoptotic extrusion [9,31]. This reinforces the notion that active neighbor cell responses are a critical part of the apoptotic extrusion phenomenon.

How does the apoptotic cell induce extrusion? Apoptotic cells undergo both chemical and mechanical changes that may trigger the extrusion process. Apoptotic cells produce a wide range of chemical signals that have often been implicated in aspects of efferocytosis [32]. Of these signals, the soluble lipid sphingosine-1-phosphate (S1P) has also been implicated in apoptotic extrusion. Immunostaining revealed that S1P was initially evident in the apoptotic cells and later appeared to accumulate in their neighbors [20]. Furthermore, extrusion was blocked by inhibiting sphingosine-1-kinase, the enzyme responsible for synthesis of S1P [20]. This suggested that S1P can be released from apoptotic cells to trigger extrusion.

Apoptotic cells also change their mechanical properties. Cells become hypercontractile when they undergo apoptosis [15,33]. In many, but not all [21], cases, this is thought to be due to the activation of procontractile kinases by apoptotic caspases. For example, Rho Kinase (ROCK) is autoinhibited by an intramolecular interaction between its N-terminus and its C-terminus [34]. However, the N-terminus of ROCK1 is cleaved by caspase-3, yielding a fragment that can constitutively activate downstream contractile signaling [15,33], to promote Myosin II and LIM kinase activation. Apoptotic epithelial cells show features of hypercontractility, such as blebbing and an increase in cortical actomyosin [9,11,15,21,33]. Conversely, the sporadic expression of truncated, constitutively active ROCK2 mutants in epithelia can induce extrusion, even though the transgene-expressing cells do not undergo apoptosis [9,11]. Together, these observations imply that enhanced contractility within apoptotic cells can provide a mechanical stimulus for extrusion.

How do neighbor cells expel apoptotic cells? To address this question, we focus on the mechanical processes that allow neighbor cells to expel apoptotic cells and their underlying cell biological mechanisms. Two mechanisms are currently known that can allow neighbor cells to apply compressive forces onto, and therefore expel, dying, apoptotic cells. 

First, the contractile cortex can be reinforced within neighbor cells, especially at their interface with the apoptotic cell. Both F-actin and Myosin II have been observed to accumulate at this site during the extrusion process. This generates a cup-like actomyosin network with increased contractile tension [11] within the neighbor cells specifically at its junction with the apoptotic cell. Contraction in this enhanced actomyosin cortex could effectively apply compressive forces to expel the apoptotic cell. It should be noted that in this model, apical extrusion would imply that neighbor cell contraction is principally applied at their basal regions, creating a net compression that directs the apoptotic cell apically. Indeed, this has been observed in many cases [9,10,11].

In the second mechanism, compression can be generated by the lamellipodial protrusions that neighbor cells make as they crawl underneath the apoptotic cells [35]. It has been suggested that cortical contractility may operate for single cells, whereas lamellipodia may be involved where larger cells, or small groups of cells, are being extruded [29]. These mechanisms are not necessarily exclusive, as cryptic lamellipodia which extend underneath migrating epithelial cells can also form AJ [36,37]. 

Both of these processes imply that the cytoskeleton is altered within neighbor cells in response to signals from the apoptotic cell. This would entail intracellular signaling pathways in the neighbor cell that ultimately regulate the cytoskeleton, especially the actomyosin apparatus. To date, the RhoA GTPase, a canonical activator of the actomyosin cytoskeleton, is the signal that has been most extensively studied in this process. Levels of the active GTP-loaded form of RhoA have been reported to increase in neighbor cells and selective inhibition of RhoA by microinjection of C3-transferase into the neighbor cells blocked apoptotic extrusion [2,9]. RhoA is activated by guanine nucleotide exchange factors (GEFs) and, consistent with this, p115 RhoGEF also localized to the basal regions of neighbor cells during apoptotic extrusion [10]. Many other cytoskeletal effectors and signals are involved in both cell contractility [11] and lamellipodial activity, so it is likely that other important pathways remain to be identified.

**Figure 2:**
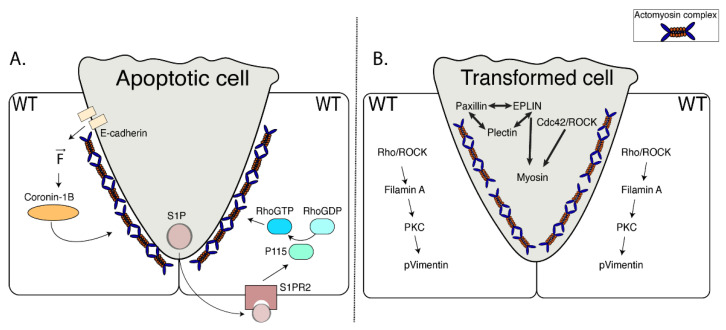
Types of extrusion. (**A**) Apoptotic cell extrusion. There are two known pathways for apoptotic cells to elicit extrusion responses in their nonapoptotic neighbors. [Left side] When a cell undergoes apoptosis, it becomes hypercontractile. This contractile force is sensed by its healthy neighbors through E-cadherin which leads, in turn, to assembly of an actomyosin complex through coronin-1B. [Right side] The apoptotic cell releases S1P. S1P binds to S1P_2_ receptors present on the healthy neighbors of the dying cell; this ultimately activates RhoA through a signal transduction pathway that involves p115 RhoGEF. The active form of RhoA mediates assembly of actomyosin complex in the neighbors. (**B**) Oncogenic cell extrusion. Depending on the type of oncogene expressed, a transformed cell elicits distinct signaling pathways which potentially converge to generate biomechanical changes. Activation of the Cdc42/ROCK signaling pathway and upregulation of cytoskeletal-interacting proteins (e.g., EPLIN, paxillin, and plectin) enhance contractility in the transformed cell. Simultaneously, in the neighboring wild-type cells, activation of the RhoA/ROCK signaling pathway upregulates other cytoskeletal proteins (e.g., Filamin A, vimentin). Mechanisms triggered in both the transformed and wild-type cells display mutual coregulation.

Importantly, there are pathways for these chemical and mechanical apoptotic signals to elicit the cytoskeletal responses of their neighbors. Extrusion would then arise from intercellular communication between these two cell populations (Figure 2A).

The capacity for cell-to-cell communication is best understood for S1P, where extrusion could be blocked by antagonizing the S1P receptor specifically in the neighbor cells [20]. Of the four classes of S1P receptors present in vertebrates (S1P_1_ to S1P_4_), the S1P_2_ receptor appears to be specifically involved, as extrusion was blocked with antagonist drugs and RNAi specific for this molecule [20]. However, extrusion was not inhibited by depleting S1P_2_ in the apoptotic cell itself [20], which made it unlikely that S1P was working in an autocrine fashion. Instead, extrusion was blocked when S1P_2_ was depleted in the surrounding epithelium [20]. This implied that the S1P_2_ receptor was only required in the neighbor cells, yielding a paracrine model where S1P secreted by the apoptotic cell binds to S1P_2_ on its neighbors to elicit the extrusion response (Figure 2A). As S1P_2_ is a G-protein coupled receptor that can activate p115 RhoGEF via its associated Gα12/13 subclass of G-proteins [10], it could anchor a paracrine pathway to ultimately activate actomyosin in the neighbor cells via RhoA.

Adherens junctions also provide the potential for mechanical signals from apoptotic cells to be transmitted to their neighbors. Indeed, a number of tension-sensitive mechanisms have recently been identified to associate with E-cadherin [38,39,40,41,42] which could respond to the enhanced contractility of apoptotic cells. They would also explain why apoptotic extrusion requires E-cadherin to be present in the neighbor cells. Interestingly, one of these, which utilizes Myosin VI as a mechanosensor [42], also activates RhoA via p114 RhoGEF, a close relative of p115 RhoGEF. Although tantalizing, though, a role for mechanotransduction has yet to be tested in apoptotic extrusion.

## 4. Oncogenic Cell Extrusion

Cell extrusion also occurs when oncogenes are sporadically expressed in epithelia [43,44,45,46]. In vertebrates, oncogenic extrusion commonly occurs in the apical direction, although basal extrusion has also been observed [3,47,48,49] (Figure 2B). Oncogenic extrusion can be elicited in cultured epithelial monolayers [3,45,50,51,52,53], indicating that it is an epithelium-intrinsic process like apoptotic extrusion. Extrusion typically occurs only when oncogenes are expressed in single cells or small groups of cells that are surrounded by nonexpressing epithelium. Indeed, extrusion did not occur when transforming Ras mutants were expressed ubiquitously in MDCK monolayers [3].

Extrusion can be elicited by a diverse range of oncogenes. In addition to Ras, these include Src [54], Cdc42 [55], Yap1 [56], and ERBB2 [46]. In addition to a range of cultured epithelia, oncogenic extrusion occurs in Drosophila [3], zebrafish [57], and mouse models [58]. Thus, it has been suggested that extrusion represents a fundamental response of epithelia to early transformation. The functional consequence of extrusion is less clear. One possibility is that apical extrusion represents a mechanism to eliminate newly transformed cells from the body (and it has accordingly been described as an epithelial defense against cancer, EDAC) [59,60,61]. Akin to apoptotic extrusion, apical extrusion would be considered to expel newly transformed cells into the external environment. However, apical extrusion may also allow transformed cells to proliferate, by removing them from inhibitory influences in the host epithelium [46], something that may be especially relevant where the apical compartment is relatively enclosed. It should also be noted that experimental studies to elicit extrusion have typically expressed strong oncogenes. It will be interesting to test whether extrusion is elicited or altered by the loss of tumor suppressor genes or as driver mutations accumulate during the natural history of cancer.

What changes in the oncogene-expressing cells? The oncogenes tested to date include small GTPases (Ras) and tyrosine protein kinases (e.g., Src, ERBB2) that are capable of engaging a diverse range of downstream signaling pathways. Therefore, it is necessary to identify the pathway(s) and their effector targets that are responsible for eliciting extrusion.

Indeed, a variety of signaling pathways are activated in cells that mosaically express oncogenes such as Ras or Src, but their relative contributions can differ. For example, Cdc42 activity increases in cells that express constitutively active H-Ras^V12^ and extrusion is inhibited by coexpression of a dominantly interfering Cdc42 mutant [3]. Furthermore, expression of a constitutively active Cdc42 mutant could itself induce extrusion [55]. Thus, Cdc42 appears to be a key mediator of extrusion downstream of Ras. However, ERK/MAPK activity also appeared to be necessary for Ras to induce extrusion, although expression of constitutively active Raf, which mediates signaling from Ras to ERK, did not induce extrusion by itself [3]. Therefore, although signals like Cdc42 may play critical roles in oncogene expressing, extrusion is likely to reflect their interaction with other signaling pathways.

A number of potential effector targets have also been identified in oncogene-expressing cells, whose effects can provide insight into the mechanism of cell expulsion. For example, levels of EPLIN, plectin, and paxillin were upregulated in cells mosaically expressing H-Ras^V12^ and these appeared to have secondary impacts on membranes and the cytoskeleton [50,51,52,57,59]. EPLIN appeared to upregulate phosphomyosin levels while paxillin and plectin were implicated in increasing tubulin and acetylated tubulin levels in H-Ras^V12^ cells, perhaps by inhibiting the α-tubulin deacetylase, HDAC [51,62]. Interestingly, atomic force microscopy measurements suggested that H-Ras^V12^ cells become stiffer and more viscous [3], but whether these cytoskeletal changes are responsible for that mechanical change remains to be tested. It might also be anticipated that apical extrusion would require the de-adhesion of cells from their underlying extracellular matrix. Thus, it is interesting that matrix metalloproteinase expression is increased in cells that mosaically express constitutively active Cdc42 or ERBB2 [46,55]. Interestingly, MMP inhibition reduced the extrusion of ERBB2-expressing cells and, indeed, mosaic overexpression of MT1-MMP could itself support extrusion. Although the picture remains incomplete, these findings suggest that multiple cytoskeletal and adhesive changes occur in oncogene-expressing cells which collaborate to support extrusion.

How do the neighbor cells change? In contrast, it is much less clear how neighbor cells are altered during oncogenic extrusion. Whereas an enhanced actomyosin cortex is commonly seen in neighbor cells during apoptotic extrusion, it has seldom been observed during oncogenic extrusion [3]. Even though RhoA/ROCK signaling was required in neighbor cells, myosin activity was dispensable [59]. Thus, oncogenic extrusion does not appear to require a consistent contractile response in the neighbor cells, as is the case for apoptosis. Instead, other cytoskeletal changes have been reported to occur in the neighbors of oncogene-expressing cells. These include upregulation of filamin, an F-actin cross-linker which promotes cell surface tension [63], whose accumulation in neighbor cells depends on RhoA/ROCK signaling [59]. As well, intermediate filament components are upregulated in neighbor cells [59]. Keratin 5/8 was enhanced around H-Ras^V12^ cells and vimentin accumulated in the neighbors of v-Src cells. Intermediate filaments are capable of strain stiffening, to support the mechanical integrity of cells and couple to desmosomes to promote cell–cell adhesion and tissue integrity [64,65]. However, how this may contribute to the extrusion of oncogene-expressing cells is not yet clear, and its elucidation is likely to require a clearer understanding of the mechanical events that drive oncogenic extrusion (see below).

Is there cell–cell communication in oncogenic extrusion? Are these neighbor cell changes occurring in response to signals from the oncogene cell? The answer to this question is much less clear than that for apoptosis. The S1P receptor, S1P_2_, has been reported to be required in neighbor cells for H-Ras^V12^-induced extrusion [47,48,61], but whether this is responding to an S1P signal from the H-Ras^V12^ cells is unclear. Indeed, extrusion was not affected by inhibiting cellular production of S1P by sphingosine-1-kinase [61]. As well, S1P levels were reduced by autophagy when K-Ras^V12^ cells underwent basal extrusion [48].

Further, the nature of cell-to-cell signaling appears to be much more complex for oncogenic extrusion than is the case for apoptotic extrusion. For example, active GTP-Cdc42 levels were increased in cells that mosaically expressed H-Ras^V12^ (i.e., those whose neighbors did not express Ras), but not when Ras was ubiquitously expressed in the monolayers [3]. Thus, oncogene-free neighbors were necessary for Cdc42 to be activated in the H-Ras^V12^ cells. Similarly, ephrin-A in neighbor cells has been reported to activate its cognate receptor, EphA2, in H-Ras^V12^ cells, to support F-actin and Myosin II levels in the oncogene-expressing cells [66,67]. Together, these observations imply that there is signaling cross-talk between both oncogene cells and their oncogene-free neighbors that contributes to the nonautonomous nature of this extrusion phenomenon.

## 5. Thoughts for the Future

So, where does this leave us in our efforts to identify commonalities between apoptotic and oncogenic extrusion? Overall, we appear to have a reasonable working model to help guide investigation of apoptotic extrusion (Figure 2A). Here, signals from the apoptotic cell elicit cytoskeletal responses in its neighbor cells that ultimately generate compressive forces that expel the apoptotic cell from the epithelium. These apoptotic signals may be chemical and/or mechanical and the cytoskeletal responses in the neighbors can be contractile and/or lamellipodial. It will therefore be important to test if these options are alternatives that are used in different contexts or may function as complementary pathways. Indeed, considering the second possibility, it is interesting to consider how a diffusible apoptotic signal, such as S1P, can elicit a localized contractile response in the immediate neighbors of the apoptotic cell [2,11,20]. One possibility is that mechanotransduction cooperates with S1P to refine the site where RhoA is activated. Nonetheless, even with these open questions, the current picture provides a framework to understand why apoptotic extrusion is a cell nonautonomous phenomenon. It also begins to explain how the cell biological changes in apoptotic cells and neighbors account for the biomechanical process of extrusion.

In contrast, a unifying model for oncogenic extrusion is less evident on present evidence (Figure 2B). In particular, despite the wealth of biochemical changes that have been documented to occur in both the oncogene-expressing cell and its neighbors, the mechanics of oncogenic extrusion have yet to be characterized in sufficient depth to explain how these biochemical changes lead to the morphogenetic event of extrusion. Tissue mechanics do affect the efficacy of oncogenic extrusion. For example, at the interface between epithelial cells, mechanical tension is greater at the apical zonula adherens (ZAs) than in the lateral cell–cell contact surfaces found basal to the ZA [53]. However, increasing lateral tension at the cell–cell junctions compromised extrusion. On a larger length scale, increasing tension within epithelial monolayers also antagonizes extrusion [68].

However, to understand how these hypertensile changes exert their impact, we will need to understand the mechanobiology of oncogenic extrusion. It seems likely that expulsion of oncogene-expressing cells requires compression from its surrounding epithelium, but this has to be confirmed experimentally, and then we need to elucidate the responsible cellular mechanism. In particular, it will be important to understand whether and how the mechanical changes of extrusion arise from the biochemical cross-talk that occurs between oncogene cells and their neighbors. This knowledge will help us compare these two forms of extrusion and better define their commonalities and differences. It will also provide a basis to characterize the diverse other forms of extrusion that have been documented.

Finally, it will be important to understand the degree to which epithelial integrity is preserved in different kinds of extrusion and how this is accomplished. As noted above, apoptotic extrusion preserves the epithelial barrier. How this may be coordinated with the process of apoptotic corpse expulsion is less clear, but it is interesting to consider whether the contractile forces of apoptotic extrusion coordinate the assembly of junctions between neighbors. Both contractility within apoptotic cells [2] and cortical contraction in the neighbors [11] could help to bring the surfaces of neighbor cells together, zippering them up as the apoptotic cell is expelled. In contrast, whether the epithelial barrier is preserved during oncogenic extrusion has not been thoroughly analyzed. Junctional proteins, such as E-cadherin and β-catenin, have been reported to redistribute to the interface between oncogene-expressing cells and their neighbors [54,55,57], a process that may involve cell contractility [54]. But it will be important to test directly whether or not the epithelial barrier remains intact, as has been demonstrated for apoptotic extrusion [2], and, if so, how interactions between neighbors are preserved to achieve this. Alternatively, if the epithelial barrier is not preserved during oncogenic extrusion, this might be another clue to suggest that it is biologically distinct from apoptotic extrusion.
